# Supplementary material for: Understanding COVID-19 Epidemiology and Implications for Control: The Experience from a Greek Semi-Closed Community
Source: J Clin Med. 2021 Jun 23;10(13):2765. doi: 10.3390/jcm10132765 (PMC8268522; doi:10.3390/jcm10132765)
Supplement: Supplementary file 1 [file jcm-10-02765-s001.zip › jcm-1230423-supplementary.pdf]

HELLENIC REPUBLIC
MINISTRY OF HEALTH & SOCIAL SOLIDARITY
GENERAL UNIVERSITY HOSPITAL OF LARISSA
LABORATORY OF MICROBIOLOGY
HEAD: Professor Euthimia Petinaki

Date: 29/1/2021

Clinical evaluation of the performance of the Lateral Flow Assay [V1301 -V1310-V1330] Rapid Test Ag 2019-nCoV, from ProGnosis Biotech SA that directly detect antigens of SARS-CoV-2

To whom it may concern:

This letter serves as verification of the Clinical Evaluation Study that took place on the period of 2nd of December 2020 until the 29th January 2021 at the Laboratory of Microbiology of the General University Hospital of Larissa, which also serves as one of the official Laboratories for SARS-CoV-2 in Greece during the pandemic of COVID-19.

The purpose of this study was the Clinical Evaluation of the **Lateral Flow Assay Test [V1301-V1330] Rapid Test Ag 2019-nCoV** produced from **ProGnosis Biotech SA**, that directly detect antigens of severe acute respiratory syndrome coronavirus 2 (SARS-CoV-2).

The samples used for the conduction of the Clinical Evaluation on the following report were positive and negative samples confirmed with RT-PCR at the **Laboratory of Microbiology of the General University Hospital of Larissa.** Both the negative and positive samples were assayed on **RT-PCR using SARS-COV-2 R-GENE® Biomerieux, RNeasy Mini Kit Qiagen & Vircell SARS-COV-2 REALTIME PCR KIT** and on the **Lateral Flow Assay using [V1301-V1330] Rapid Test Ag 2019-nCoV ProGnosis Biotech SA.**

ΕΦΗ ΠΕΤΕΙΝΑΚΗ
ΚΑΘΗΓΗΤΡΙΑ ΙΑΤΡΙΚΗΣ ΒΙΟΠΑΘΟΛΟΓΙΑΣ-ΚΛΙΝΙΚΗΣ ΜΙΚΡΟΒΙΟΛΟΓΙΑΣ
ΔΙΕΥΘΥΝΤΡΙΑ ΕΡΓΑΣΤΗΡΙΩΝ ΜΙΚΡΟΒΙΟΛΟΓΙΑΣ [illegible]
ΚΑΙ ΚΛΙΝΙΚΗΣ [illegible]
Π.Γ.Ν. ΛΑΡΙΣΑΣ

Prof. Efi Petinaki
Head of Department of Microbiology
University of Thessaly
Larissa, Greece

Tel: 2413502517, 2413502808 Fax: 2413501570350, Email: petinaki@med.uth.gr

1. Protocol synopsis:

**Title**	Evaluation of lateral flow assay tests that directly detect antigens of SARS-CoV-2 and can be interpreted visually
**Short title**	COVID-19 Antigen **Rapid Diagnostic Test (RDT)** Evaluation
**Use case of test**	Rapid, point-of-care (POC) detection of active infection in adults with suspected COVID-19 infection.
**Rationale and background**	The aim of this study is to independently evaluate the performance of a novel, rapid, **point-of-care (POC)** lateral flow assay developed and produced by **ProGnosis Biotech SA**, for the direct detection of SARS-CoV-2 antigens (Ag) in comparison to the current gold standard(method) for testing, RT-PCR.<br>**This protocol covers the approach to assess the performance of index RDTs:**<br>• A retrospective clinical approach using respiratory swabs collected in assay-specific buffer from individuals confirmed or suspected COVID-19, as defined by national or WHO case definitions.<br>• If rapid diagnostic tests (RDTs) that detect SARS-CoV-2 antigen are shown to have sufficient accuracy and sensitivity, then their use could facilitate rapid clinical decision making, as these tests are very simple to perform and the turnaround time for results is typically < 30 minutes
**Primary objective(s)**	[Clinical Evaluation] To determine the diagnostic accuracy of COVID-19 antigen RDT in patients using upper respiratory tract specimens compared to gold-standard method (RT-PCR).
**Exploratory objective(s)**	To assess the feasibility, of the index test (NP swabs and processing with RDT)
**Study design**	**Clinical Evaluation:** This is a **retrospective , performance evaluation** study of a SARS-CoV-2 Ag RDT. All index test results are compared to RT-PCR results, are for research use only, and will not be reported for patient care.<br><br>Briefly, at least 100 COVID-19 PCR positive remnant, archived remnant swab samples and at least 200 COVID-19 PCR negative remnant, archived remnant swab samples should be assessed per test; operators will be blinded to sample reactivity.
**Index Test**	V1301/ V1302/ V1310/V1330 Rapid Test Ag 2019-nCov from Prognosis Biotech SA that detect antigens of the SARS-CoV-2 virus within 15 min
**Reference test(s)**	RT-PCR (lab validated, site-specific)
**Study Samples**	**Clinical Evaluation**: (retrospective) samples sources from de-identified, remnant swab specimens which have been collected from individuals suspected to have COVID19.<br>All samples will have documented RT-PCR results. If possible, a range of samples across days from symptom onset and severity of symptoms should be included.

**Sample size**	A minimum of 50 COVID-19 RT-PCR positives (100 preferred); a minimum of 100 COVID-19 RT-PCR negatives (200 preferred)
**Ethics**	All clinical studies will be performed on samples in which individuals provided informed consent for additional or archived/remnant samples to be used for research purposes.

2. Product Info:

**Manufacturer Name**	Prognosis Biotech SA
**Test name**	Rapid Test Ag 2019-nCoV
**Product Code(s)**	V13
**Pack size(s)**	1 / 2 / 10 / 30 tests / kit
**Contents of kit**	Tests with desiccant in a pot, Buffer, Extraction tubes, positive control, negative control, sample collection swabs, quick reference guide, IFU
**Equipment and consumables required, but not provided**	PPE, Timer, Biohazard container
**Product Storage (temperature range)**	2-30°C
**Shelf-life (months)**	12 months
**Manufacturing Site (country)**	Greece

3. Study details:

**Clinical Study Design:**	Prospective diagnostic evaluation studies across multiple, independent sites to determine the accuracy of COVID-19 antigen RDTs, using consecutive enrolment. Interim analyses are performed at 25% and 50% enrolment, and the evaluation is stopped if tests do not meet 97% specificity. Presence of symptoms, date of symptom onset and hospitalization status is collected for all enrolled participants.
**Index assays:**	Novel lateral flow format tests that detect recombinant SARS-CoV-2 antigens.
**Reference method:**	Results of the index test are compared to the routine, diagnostic RTPCR result, which is used for clinical management
**Clinical**	**Sensitivity** was calculated as the proportion of true positive results detected by Rapid Test Ag 2019-nCoV among all positives by the reference method, and reported as a percentage.

Performance:	**Specificity** was calculated as the proportion of true negative specimens, identified as negative by Rapid Test Ag 2019-nCoV among all negatives by the reference method, and reported as a percentage. The 95% confidence intervals were calculated in order to assess the level of uncertainty introduced by sample size, using the Wilson's score method.

4. Evaluation Details:

**Country of Collaborator**	Greece
**Location of clinical site(s) (city, town)**	University Hospital of Larissa, Larissa, Thessaly, Greece
**Health care level of site(s)**	Emergency Department
**Study period (date to date)**	December 2020 -January 2021
**Sample type, antigen test**	Nasopharyngeal swab
**Reference PCR Method**	Genesig Primer design Coronavirus (COVID-19) CE IVD, Vircell SARS-COV-2 REALTIME PCR KIT
**Sample type, PCR test**	Nasopharyngeal / oral swab

5. Results:

Table 1

**Rapid Test Ag 2019-nCoV**	**Real-time RT PCR**		
	Positive	***Negative***	***Total***
Positive	105	260	370
Negative	4	1	
Total	109	261	

ΕΦΗ ΠΕ[illegible]ΙΝΑΚΗ
ΚΑΘΗΓΗΤΡΙΑ ΙΑΤΡ[illegible] ΒΙΟΠΑΘΟΛΟΓΙΑΣ-
ΚΛΙΝΙΚΗΣ [illegible]ΒΙΟΛΟΓΙΑΣ
ΔΙΕΥΘΥΝΤΡΙΑ ΕΡΓΑΣΤΗΡΙ[illegible]Ν ΜΙΚΡΟΒΙΟΛΟΓΙΑΣ
ΚΑΙ ΚΛΙΝΙΚΗΣ [illegible]ΙΕΙΝΗΣ
Π.Γ.Ν. ΛΑΡΙΣΑΣ

6. Estimations of Clinical Performance

- ***All Samples included Performance Characteristics***

Table 2

	Mean Value	95% confidence interval
Sensitivity	96.33%	90.87% to 98.99%
Specificity	99,62%	97.88% to 99.99%
Positive Predicted Value	99,06%	93.69% to 99.87%
Negative Predicted Value	96.49%	96.13% to 99.42%
Accuracy	98.65%	96.87% to 99.56%

Table 3

CT cycles	RT-PCR positive	Rapid Test Ag 2019-nCoV positive	Positive Agreement (95% CI)
<21	1	1	100.00% (54.07% to 100.00%)
22-25	8	8	100.00% (63.06% to 100.00%)
26-29	43	41	95.83% (85.75% to 99.49%)
30-33	52	52	100% (93.15% to 100.00%)
>34	3	1	33.33% (0.84% to 90.57%)

- Only samples up to 33 PCR cycle included Performance Characteristics.

Table 4

	Mean Value	95% confidence interval
Sensitivity	98.08%	93.23% to 99.77%
Specificity	99.62%	97.88% to 99.99%
Positive Predicted Value	99,03%	93.51% to 99.86%
Negative Predicted Value	99.24%	97.05% to 99.81%
Accuracy	99.18%	97.62% to 99.83%

ΕΦΗ Π[illegible]ΙΝΑΚΗ
[illegible]
Π.Γ.Ν. ΛΑΡΙΣΑΣ
